# One-year follow-up after treatment of proximal and/or middle one-third humeral shaft fractures with a helical plate: healing rates, complications and functional outcome measures

**DOI:** 10.1186/s12891-021-04774-9

**Published:** 2021-10-20

**Authors:** V. Maes, G. Putzeys

**Affiliations:** 1 Department of Orthopaedic Surgery, University Hospitals Leuven, Leuven, Belgium; 2 Department of Trauma and Orthopaedic Surgery, AZ Groeninge, Kortrijk, Belgium

**Keywords:** Helical plate, Diaphyseal humeral fracture, Humeral shaft fracture, Radial nerve palsy, Functional outcome

## Abstract

**Background:**

Conventional plate osteosynthesis is a valuable treatment option in displaced proximal and/or middle one-third humeral shaft fractures. Nonetheless, this procedure can be complicated by a radial nerve palsy. To date, many surgical techniques have been developed in an attempt to minimize this high-impact complication. A helical plate has the potential to avoid an iatrogenic radial nerve palsy due to its design. This article aims to evaluate safety and functional outcomes of patients treated with a helical plate compared to conventional plate osteosynthesis. In particular healing rates, complications and functional outcome measures.

**Methods:**

We retrospectively included all patients with displaced proximal and/or middle one-third humeral shaft fractures who were treated with a helical plate from October 2016 until August 2018 at a single level-1 trauma center (AZ Groeninge, Kortrijk, Belgium). A self-molded long PHILOS plate (DePuy Synthes®) or a pre-contoured A.L.P.S proximal humeral plating system (Zimmer Biomet®) were used. Patient baseline characteristics and standard radiographs were obtained pre- and postoperatively. We retrospectively searched for complications. Patients were reassessed using the Disabilities of the Arm, Shoulder and Hand (DASH), Constant Murley (CMS) and EQ-5D-5L scores with a minimal follow-up of 1 year.

**Results:**

The humeral shaft fractures of all sixteen patients consolidated within 3 months and no iatrogenic radial nerve palsies were observed. One plate had to be removed after 1 year due to a late deep infection. With a minimum follow up of 1 year, the mean DASH score was 22 ± 19 and the mean normalized CMS was 80 ± 19.

**Conclusion:**

Operative treatment of proximal and/or middle one-third humeral shaft fractures with a helical plate is a safe procedure with good to excellent shoulder function at one-year follow-up. Contrary to conventional plate osteosynthesis, a helical plate has the potential to completely avoid a radial nerve palsy, while maintaining similar healing rates and functional outcomes.

**Trial registration:**

Retrospective cohort study. B396201939564. Registered on 10 MAY 2019.

**Supplementary Information:**

The online version contains supplementary material available at 10.1186/s12891-021-04774-9.

## Background

### Introduction

Humeral shaft fractures, or diaphyseal humeral fractures, represents 1-3% of all fractures [1]. The majority, approximately 84%, consists of proximal and/or middle one-third humeral shaft fractures [1].

Although humeral shaft fractures are generally treated conservatively, surgery is a valuable treatment option in displaced fractures as it reduces the risk of non-union and leads to faster restoration of daily activities [2, 3]. Plate osteosynthesis is preferred to intramedullary nailing in humeral shaft fractures with proximal extension as the latter increases the risk of shoulder impingement, restriction of shoulder movement and need for removal of metalwork [4]. Furthermore, anatomic reduction could be attempted with plate osteosynthesis. Angular deformity can thus be corrected in order to maximize cortical contact. Nonetheless, radial nerve palsy (RNP) is a serious complication following conventional plate osteosynthesis [5]. A meta-analysis of 2020 comparing conservative versus operative treatment of humeral shaft fractures, showed a 3,5% RNP complication in the operative treatment group [2]. However, this percentage is the result of a combination of different operative treatment modalities. A recent meta-analysis regarding open reduction internal fixation (ORIF) versus minimally invasive plate osteosynthesis (MIPO) reported an occurrence of 8,3% of iatrogenic RNP in the ORIF group [6]. Furthermore, iatrogenic RNP is reported up to 22%, depending on the surgical approach used [7]. This iatrogenic high impact complication could be avoided with the use of a helical plate due to its design and corresponding surgical approach [8].

In 2002, Fernandéz published “The principle of helical implants”, wherein the biggest advantage of a helical implant is attributed to its ability to cover different zones in different planes of the same bone [8]. This way, the plate will cover both the lateral side of the proximal third of the humerus, avoiding the long head of the biceps, and the anterior side of the middle/distal third of the humerus, avoiding the radial nerve and deltoid insertion [8]. In other words, a helical plate has the potential to completely avoid an iatrogenic RNP and will not compromise the anterior deltoid [8, 9].

Despite the promising concept of helical plates there is still a lack of published evidence to date regarding safety and functional outcome in proximal and/or middle-one third humeral shaft fractures.

In this clinical outcome study, we are the first to report on the use of the pre-contoured A.L.P.S proximal humeral plating system (Zimmer Biomet®) and its functional outcomes after one-year follow-up.

### Objectives

To evaluate safety and functional outcomes of patients with proximal and/or middle one-third humeral shaft fractures treated with a helical plate, compared to conventional plate osteosynthesis. In particular healing rates, complications and functional outcome measures.

## Methods

### Study design

The study protocols of this retrospective cohort study adheres to the principles outlined in the Declaration of Helsinki and were approved by the institution’s ethics committee (B396201939564). A written informed consent was obtained before reassessment for functional outcomes measures.

### Setting and participants

We retrospectively and consecutively included all patients with humeral shaft fractures who were treated by ORIF with a helical plate from October 2016 until August 2018 at a single level-1 trauma center (AZ Groeninge, Kortrijk, Belgium). Indications for surgery are displaced proximal and/or middle humeral shaft fractures with proximal extension and need for humeral head fixation. In addition, less displaced fractures with persistent excessive pain despite adequate conservative treatment, are also treated operatively.

### Variables and data sources

The following patient characteristics were obtained in individual medical records: age, sex, mechanism of injury, attending surgeon, type of fracture (AO/OTA classification), helical plate type and the presence of a preoperative radial nerve palsy.

We retrospectively searched for postoperative complications, e.g., radial nerve palsy, nonunion, surgical site infection (SSI), adhesive capsulitis, loosening or failure of osteosynthesis material. A surgical site infection was classified by the Center of Disease Control and Prevention as follows [10]:

1. Superficial incisional: infection within 30 days after the operation and only involves skin and subcutaneous tissue of the incision.
2. Deep incisional: infection within 30 days after the operation if no implant is left in place or within 1 year if implant is in place and the infection seems to be related to the operation and infection involves deep soft tissue (eg, fascia, muscle) of the incision.

Functional outcome measures were, after written informed consent, prospectively obtained in the Fall of 2019. We used the Disabilities of the Arm, Shoulder and Hand (DASH) score as a patient-reported functional outcome measure and Constant Murley scores (CMS) as a clinician-measured functional outcome measure. The patient’s general health status was evaluated using the EQ-5D-5L score [11]. All measures were obtained by one Orthopaedic Surgery resident within 1 month.

Only descriptive statistical analysis was made in between groups due to the small number of patients.

### Surgical technique

The patient was set up in a beach chair position. A deltopectoral approach was used in combination with a distal anterolateral incision (including a brachial split), whether or not in continuity. A MIPO technique was not used. If minimal deltoid detachment was necessary during surgery, it was performed under direct visualization. We consistently employed a surgical support arm (TRIMANO, Arthrex®). Radial nerve exploration was not performed systematically. Fractures were reduced anatomically, if possible with the use of 3.5 mm lag screws. Bone graft was not used.

**Fig. 1:**
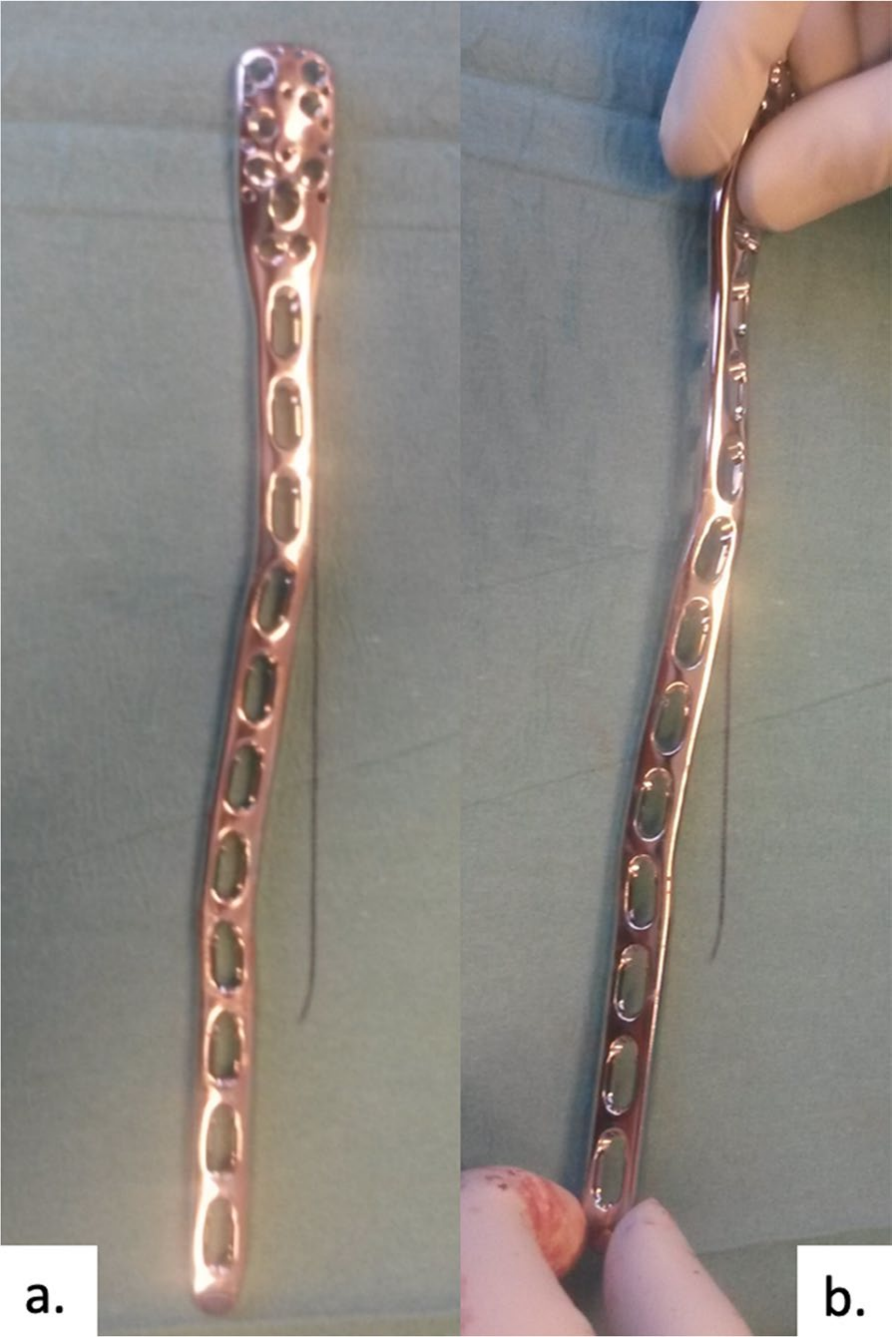
Bending of a PHILOS plate. **a** Step 1: create an S-shaped curve in the coronal plane of the middle third. **b** Step 2: rotate the distal part in about 70-90° to the proximal part

Two types of helical plates were used. A self-molded long Proximal Humeral Internal locking System (PHILOS) plate with 7, 9 or 11 shaft holes (DePuy Synthes®). The middle third of a PHILOS plate was used to make it helical. First of all, an S-shaped curve was created in the coronal plane of the middle third. Secondly, the distal part was rotated about 70-90° to the proximal part (Fig. 1).

As the second type, a pre-contoured A.L.P.S proximal humeral plating system with 11 or 14 shaft holes (Zimmer Biomet®) was used. Additional molding of the A.L.P.S. plate was performed when necessary. This plate has two different types proximally. A low plate that sits 2 cm from the greater tuberosity and a high plate that sits 1 cm from the greater tuberosity and offers two additional screw holes proximally. Parameters taken into account to decide plate length and number of screws were a minimal of three distal bicortical screws with the largest distance possible in between them. In periprosthetic fractures, unicortical screws and/or cerclage were used.

### Postoperative management

Standard radiographs, anteroposterior and lateral, of the upper arm were obtained pre- and postoperatively until union was achieved. Radiographic union was defined as the presence of callus bridging on at least three of the four cortices. In case of absolute stability (anatomical reduction), the following clinical criteria for union were used: lack of local tenderness at the fracture site and the patient’s ability to perform activities of daily living with the injured limb.

Postoperative management consisted of a removable sling for 6 weeks and early mobilization. Physiotherapy started at the earliest 4 weeks postoperative.

## Results

**Fig. 2:**
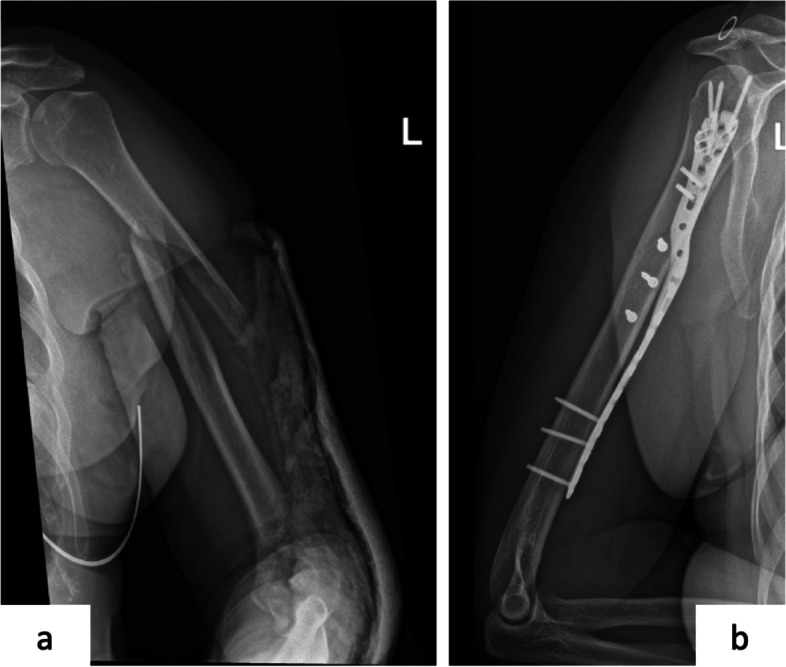
Example of a pre-contoured helical plate, female 63 years old. **a** Preoperative spiral humeral fracture (12A1). **b** Postoperative A.L.P.S. proximal humeral plating system

**Fig. 3:**
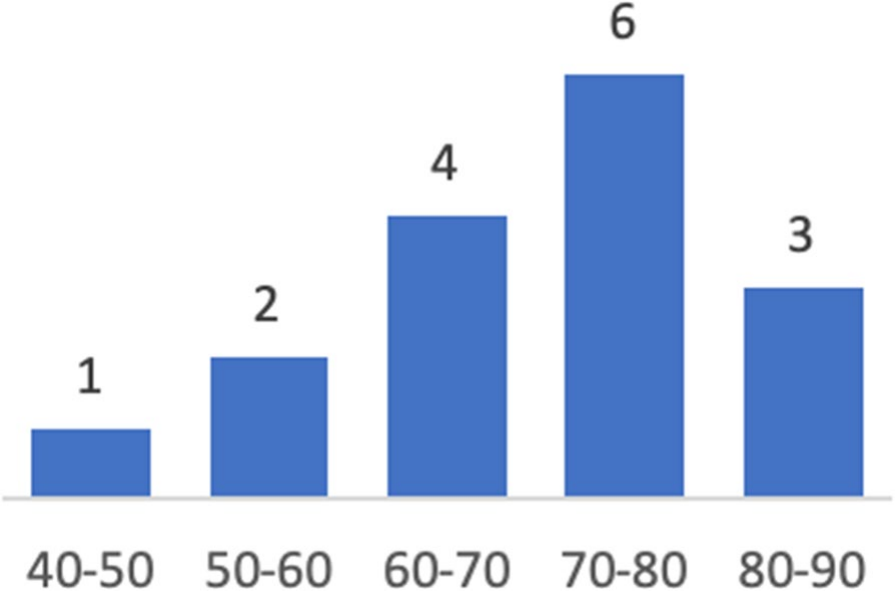
Age distribution of patient population

**Fig. 4:**
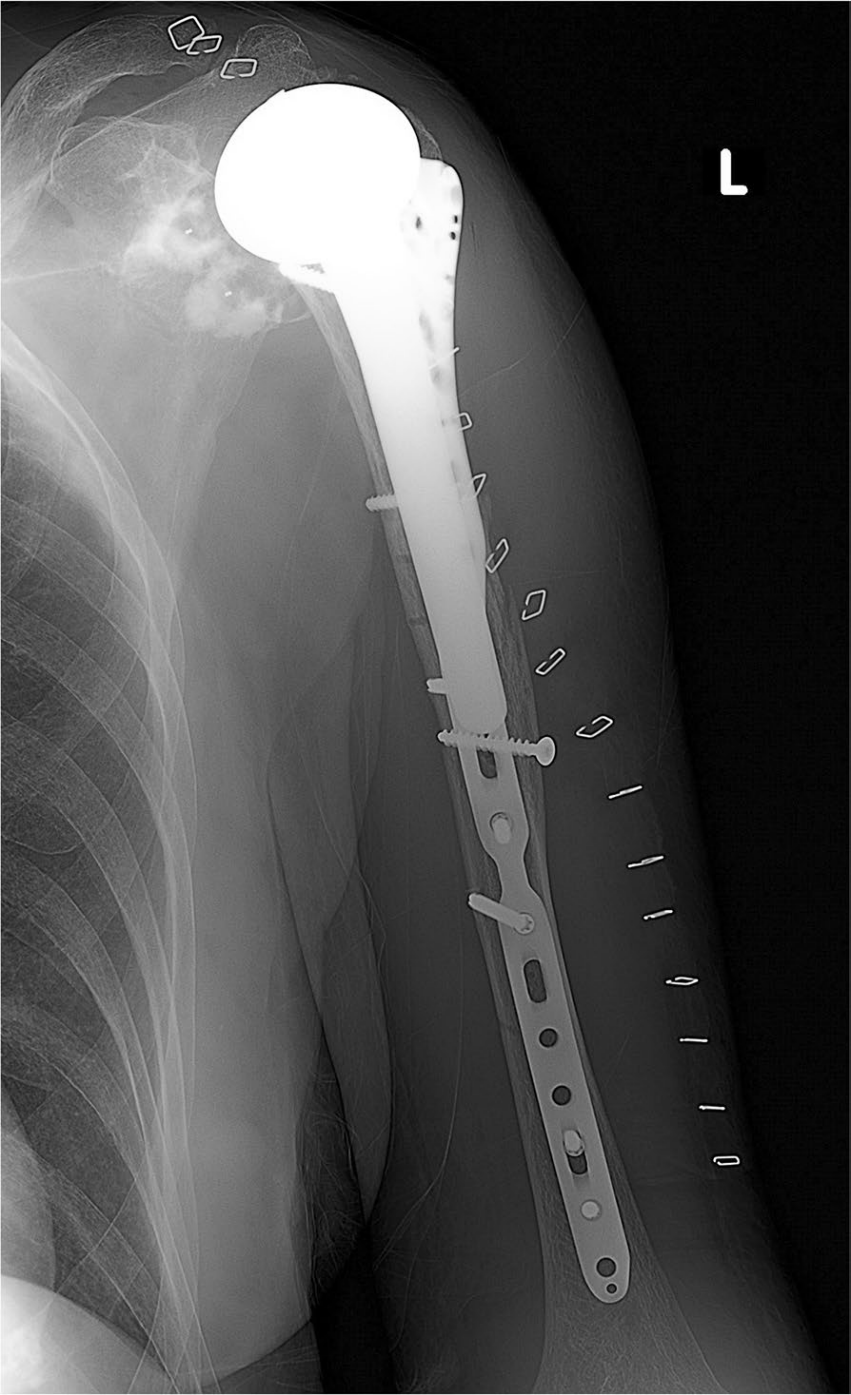
Open reduction internal fixation (ORIF) of a periprosthetic fracture with a pre-contoured helical plate, female 80 years old

Sixteen patients were treated with a helical plate between October 2016 until August 2018 at AZ Groeninge (Kortrijk, Belgium). Patient baseline characteristics can be found in Table 1. A self-molded long PHILOS plate with 7, 9 or 11 shaft holes (DePuy Synthes®) was applied in the first nine patients, while in the last seven patients the pre-contoured A.L.P.S proximal humeral plating system with 11 or 14 shaft holes (Zimmer Biomet®) was used (Fig. 2). We only employed low A.L.P.S. plates and two pre-contoured plates required additional molding. Age distribution was between 48 and 82 years old (Fig. 3)*.* All patients, except for one, were treated by the same surgeon. There was one delayed union, two periprosthetic fractures and two preoperative radial nerve palsies (Fig. 4). We only performed radial nerve exploration in one case of preoperative radial nerve palsy. The other preoperative radial nerve palsy was a delayed union, that was not intra-operatively explored due to spontaneous recovery before surgery. Both fully recovered in a period of 3 months. A second radial nerve exploration was performed with the initial use of the pre-contoured A.L.P.S proximal humeral plating system. Fractures were classified by the AO/OTA classification. Six fractures were open dissected and anatomically reduced, using the concept of absolute stability, with one to three 3.5 mm lag screws. Additionally, we performed fracture site dissection in four out of ten remaining humeral shaft fractures in an attempt to achieve anatomic reduction.

**Table 1:** Patient baseline characteristics

			Percentage %
1. Sex	Male / Female	5 / 11	31% / 69%
2. Mechanism of Injury	High energy / Simple fall (low energy)	2 / 14	13% / 87%
3. Preoperative Radial Nerve Palsy	Yes / No	2 / 14	13% / 87%
4. Fracture Type	Proximal third	7	44%
	Middle third	2	13%
	Combined proximal/middle third	5	31%
	Periprosthetic	2	13%
5. Helical Plate	Self-molded long PHILOS plate *2016-2017*	9	56%
	Pre-contoured A.L.P.S Proximal Humerus Plating System *2018*	7	44%

All fractures consolidated clinically and radiographically within 3 months. There were no iatrogenic radial nerve palsies. One self-molded plate was removed after 1 year due to a late deep SSI. In this case, fracture was exposed and reduction was difficult, however, patient’s immunity was not compromised and healing was achieved at 3 months postoperatively. Simple removal of implant was sufficient as treatment. Two patients treated with the pre-contoured helical plate developed postoperatively an adhesive capsulitis, which were treated conservatively.

After receiving a written informed consent, twelve patients were prospectively reassessed for functional outcome measures in September 2019. At this point, one patient had died and three refused due to personal reasons. The mean DASH score is 22 ± 19 and the mean CMS is 68 ± 18. Normalization of the CMS were made following Katolik L. et al [12]. The mean normalized CMS is 80 ± 19. These scores are displayed in Tables 2 and 3, detailed information is found in Supplementary File 1. Preliminary results were presented at ECTES 2019 [13].

**Table 2:** Follow-up time – AO/OTA classification – DASH – CMS – Normalized CMS

Case	Age	Sex	Follow-up (months)	AO/OTA Classification	DASH	CMS	Normalized CMS
**1**	82	M	35	12B2	13	69	78
**2**	80	F	31	Type A^3^	5	78	96
**3**	66	F	34	12A1	0	85	100
**4**	77	F	14	12C3	63	33	41
**5**	56	F	22	12C3	35	68	81
**6**	58	F	23	12B3	39	67	80
**7**	63	F	16	12A1	33	79	95
**8**	76	F	13	12A2	4	83	100
**9**	71	F	16	12C3	23	56	69
**10**	66	M	15	12A1	2	95	100
**11**	70	M	21	12B3	20	48	55
**12**	80	F	16	Type B^3^	24	55	68

^3^Periprosthetic fracture (Wright and Cofield classification)

**Table 3:** EQ-5D-5L score

M	S	A	P	AX	E
1	2	1	1	1	80
1	1	1	2	1	80
4	1	2	4	1	70
3	5	5	3	1	30
1	1	3	3	1	70
4	5	5	3	2	50
4	5	3	2	3	50
1	1	1	1	1	80
1	1	1	2	1	60
1	1	1	1	1	75
1	1	1	2	1	90
3	1	2	3	1	70

EQ-5D-5L score was divided into M (= Mobility), S (= Self-care), A (= Activity), P (= Pain), AX (= Anxiety) and E (= EQ VAS)

## Discussion

Operative treatment of displaced proximal and/or middle one-third humeral shaft fractures with a helical plate is a safe procedure with a good to excellent shoulder function at one-year follow-up. Contrary to conventional surgical techniques, we did not observe any iatrogenic radial nerve palsies while maintaining similar functional outcome measures and also obtaining excellent healing rates [2].

A helical plate could combine the benefits of anatomic reduction while also avoiding radial nerve palsy and deltoid insertion. This is obtained by the design of the plate, covering both the lateral side of the proximal third of the humerus, avoiding the long head of the biceps, and the anterior side of the middle/distal third of the humerus, avoiding the radial nerve and deltoid insertion [8]. Klepps S. et al noted that a release of more than one fifth of the anterior deltoid insertion could compromise the anterior deltoid [9].

Three nerves are at risk in ORIF of humeral shaft fractures, in particular the radial, axillary and musculocutaneous nerves. A radial nerve palsy can be caused by trauma (i.e. fracture) or is due to surgery. Clinically, it will present itself as a loss of sensation of the dorsal hand, as well as loss of active extension of wrist and fingers at the metacarpophalangeal joints [14]. Artico M. et al. outlined the surgical anatomy of the radial nerve and showed it has a consistent distance of 121 (± 13) mm between the lateral humeral epicondyle to the lateral point of crossing the posterior aspect of the humerus [15]. When using a helical plate and lag screws for anatomical reduction, bicortical screw placement from anterior to posterior should be avoided in the most dangerous zone of the radial nerve. This is located within 47,22% to 53,21% of the humeral length from the lateral epicondyle [16]. Belayneh et al. showed no statistical differences in recovery time between nonoperative and iatrogenic radial nerve palsies [17]. Mean time to recovery of a complete palsy was 25.2 weeks, and surgical intervention did not lead to faster recovery [17]. Although management of iatrogenic radial nerve palsy is mainly conservative, sometimes late exploration is necessary if there is no spontaneous recovery at three to 6 months [14]. A systematic review by Shao et al. showed a full recovery rate of 88,1%, with a mean time to recovery of 6.1 months (range 3.4 – 12 mo.) [18].

Although there is a high overall recovery rate, the rehabilitation of patients is delayed with an average of 6 months and incomplete recovery can necessitate tendon transfers [19]. These arguments show that a radial nerve palsy is a high impact complication. One which could be completely avoided with the use of a helical plate due to its design, a benefit consistently confirmed in all case reports available [3, 20–22]. Recently, Da Silva et al. reported a 10-year retrospective study of 62 patients where no radial nerve damage was reported in the helical plate group [20]. This contrasts to conventional ORIF where there are approximately 8,3% iatrogenic radial nerve palsies [6]. Streufert B. et al. recently reported 12,2% iatrogenic RNPs with ORIF on 261 humeral shaft fractures [23]. Furthermore, this percentage of iatrogenic RNP can depend on the surgical approach used. Claessen et al. reported an iatrogenic radial nerve palsy of 22% when using a lateral approach, 4% with an anterolateral approach and 11% with a posterior approach [7]. When using a helical plate for proximal and/or middle humeral shaft fractures, the high-risk lateral approach is not indicated anymore. Moreover, a posterior approach is also not suitable for proximal and/or middle humeral shaft fractures with proximal extension and need for humeral head fixation [24]. Finally, a helical plate is less indicated for distal third humeral shaft fractures considering there is no need for proximal/humeral head fixation. Furthermore, the radial nerve is at risk in the distal third of the humerus as it crosses on the anterolateral aspect between the brachialis and brachioradialis [24].

The axillary nerve, on the other hand, elongates depending on plate-bone distance. Dauwe et al. demonstrated on 42 fresh frozen cadaveric humeri that a helical plate significantly lowers plate-bone distance. This could imply less risk of nerve damage due to lower axillary nerve elongation [25].

Lastly, Gardner et al. described that the musculocutaneous nerve is most at risk when using a helical plate, due to its location on the anterior side of the middle/distal humerus. However, a ‘safe zone’ can be created due to a predictable and consistent anatomic location of the musculocutaneous nerve (99% CI: 12,2-18,8 cm distal from the greater tuberosity) [26].

In our study, all sixteen humeral fractures consolidated clinically and radiographically within 3 months. A consistency in healing rates can be found in other small case reports. Combined, all reported humeral shaft fractures in literature healed when treated with a helical plate [3, 21, 22, 27, 28]. Moreover, Yang et al. recorded a 100% healing rate in ten comminuted fractures of the proximal and/or middle one-third of the humerus [27]. In traditional operative management nonunion rates range from 0 to 9% [4]. A systematic review of Beeres et al. showed a nonunion rate of 8,5% in patients treated with ORIF [6]. These excellent healing rates of a helical plate could be related to biomechanical advantages. Krishna et al. described that a helical plate had a better gap closure in oblique fractures, reduced stress shielding, absorbed tensile stress caused by torsion and had a higher screw-holding power due to different orientation of screws [29].

However, in self-molded plates it is known that excessive deformation during contouring will damage the locking mechanism and has an impact on the fatigue properties of the plate [29]. In our case series we did not experience failure of self-molded osteosynthesis. We used an S-shaped bending technique, similar to the technique described by Fernandéz in 2001 [8]. In theory, controlled manufacturing of helical plates could resolve this potential complication. In this study the pre-contoured A.L.P.S proximal humeral plating system (Zimmer Biomet®) was used and we are the first to report clinical results.

We reassessed twelve patients, after written informed consent, with a minimum of one-year follow-up. Functional outcome measures were taken and calculated by the same Orthopaedic Surgery resident. All measurements were made within 1 month to minimize intra-observer variability. Furthermore, it is known that the CMS has a high degree of reproducibility with a low intra-observer error of 3% [30]. Normalized CMS were comparable with those reported in other case reports (80 vs. 77, 88) [21, 28]. The systematic review of van de Wall et al. included two randomized controlled trials, for those treated operatively a mean DASH score of 15 was reported [2] . Brunner et al. used a PHILOS plate in 15 humeral shaft fractures with a median CMS of 74 (56-100) and a median DASH score of 34 (24-48) [31]. These results are comparable with our mean DASH score (22 ± 19) and our mean normalized CMS (80 ± 19).

It is, however, important to note some limitations. First of all, a retrospective single center study design was used with a small study population. Consequently, only descriptive statistical analysis was made in between groups.

Secondly, this study consists of inhomogeneous patient characteristics and fracture types as can be seen in Tables 1 and 2*.* Despite these differences, all fractures healed within 3 months and no radial nerve palsies were detected. On the other hand, these inhomogeneous patient characteristics can account for the large standard deviations in our functional outcome measures. Finally, functional outcome measures were taken at different follow-up times, however, a minimum of 1 year was respected.

## Conclusion

In conclusion, the treatment of proximal and/or middle one-third humeral shaft fractures with a helical plate is a safe procedure with good to excellent shoulder function at one-year follow-up. Contrary to conventional plate osteosynthesis, a helical plate could promote bone healing due to its biomechanical advantages, minimizes damage to the deltoid muscle insertion region and has the potential to completely avoid a radial nerve palsy. All of this could facilitate rapid and good functional recovery. In the future, prospective multi-center randomized controlled studies with reasonable study population are needed to confirm the benefit of helical plates and whether or not controlled manufacturing is preferred to self-molded plates.

Level of evidence: level IV. Retrospective cohort study.

## Supplementary Information

**Additional file 1.**

## Data Availability

All data generated or analysed during this study are included in this published article and its supplementary information files.

## Abbreviations

DASH: Disabilities of the Arm, Shoulder and Hand
CMS: Constant Murley scores
